# Historical overview of spinal deformities in ancient Greece

**DOI:** 10.1186/1748-7161-4-6

**Published:** 2009-02-25

**Authors:** Elias S Vasiliadis, Theodoros B Grivas, Angelos Kaspiris

**Affiliations:** 1 Orthopaedic Department, "Thriasio" General Hospital, G. Gennimata Av. 19600, Magoula, Attica, Greece

## Abstract

Little is known about the history of spinal deformities in ancient Greece. The present study summarizes what we know today for diagnosis and management of spinal deformities in ancient Greece, mainly from the medical treatises of Hippocrates and Galen. Hippocrates, through accurate observation and logical reasoning was led to accurate conclusions firstly for the structure of the spine and secondly for its diseases. He introduced the terms kyphosis and scoliosis and wrote in depth about diagnosis and treatment of kyphosis and less about scoliosis. The innovation of the board, the application of axial traction and even the principle of trans-abdominal correction for correction of spinal deformities have their origin in Hippocrates. Galen, who lived nearly five centuries later impressively described scoliosis, lordosis and kyphosis, provided aetiologic implications and used the same principles with Hippocrates for their management, while his studies influenced medical practice on spinal deformities for more than 1500 years.

## Introduction

**Figure 1:**
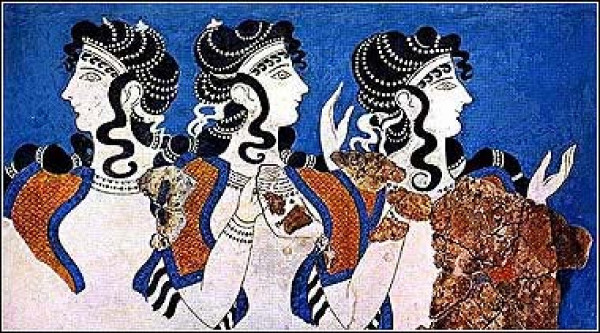
**Wall paintings of female figures wearing tight bodices that expose their breasts**. Knosos, Crete, 3^rd ^millennium BC.

**Figure 2:**
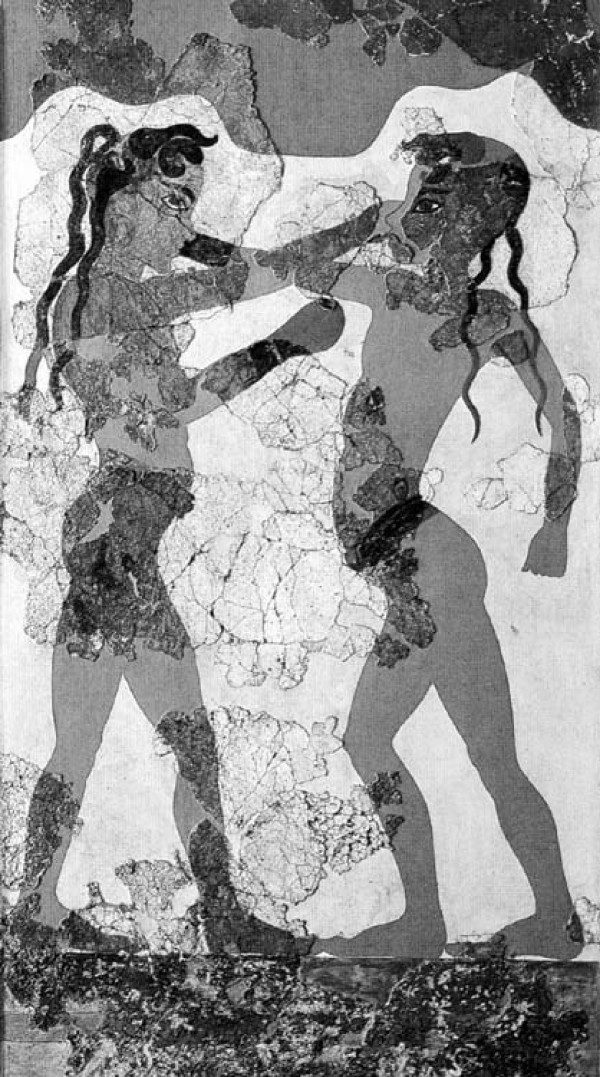
**The Boxing Boys" fresco from Room Beta 1, Akrotiri, Thera (1600 BC)**.

**Figure 3:**
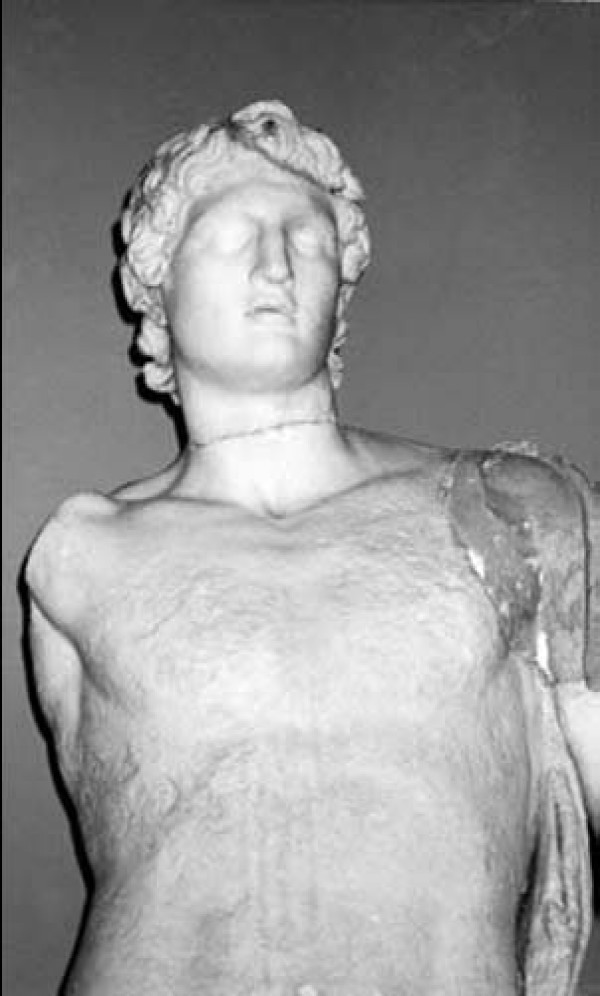
**A marble statue of Alexander the Great, showing a characteristic body asymmetry in the axis of the head, shoulders and neck, Istanbul, Turkey**.

Medicine was not clearly distinguished from religion and mysticism in the ancient world. Ancient works of philosophy, religion, myths, and fairy tales dating back as far as 3500 BC invoke images of people with spinal deformity. In the third millennium BC, wall paintings and statues from Knossos, in Crete island, depicted female figures wearing tight bodices that expose their breasts (Figure 1). Minoan Crete is considered as the origin of the corset. The *Boxing Boys *fresco (1600 BC) in Akrotiri in the Greek island of Santorini is the first monumental image of a compound spinal disorder that is diagnostically recognizable by current medical standards [1]. The painting reflects a rigid abnormality, probably a spinal deformity (Figure 2). Ancient descriptions and statues typically portray Alexander the Great with an underlying scoliotic condition with a cervical neck deformity [2], typically with a gaze looking upward and outward with the added possibility of ocular muscle deficits and facial asymmetry (Figure 3).

**Figure 4:**
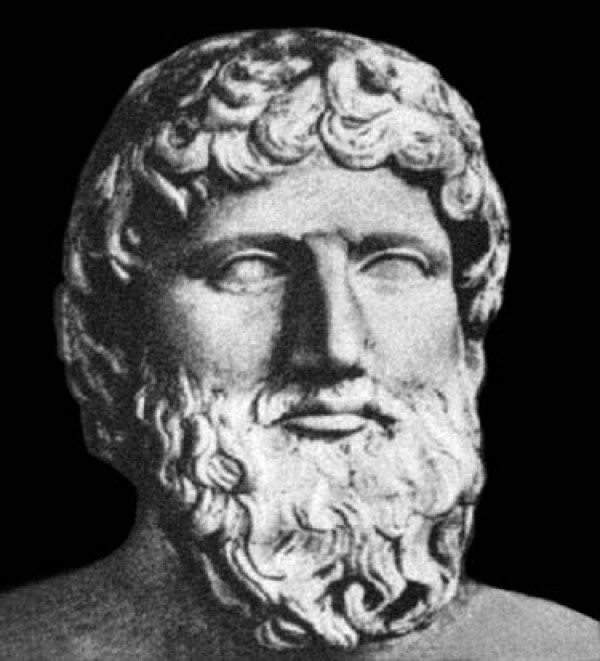
**Marble portrait of Plato, an eminent Classical Greek philosopher**.

**Figure 5:**
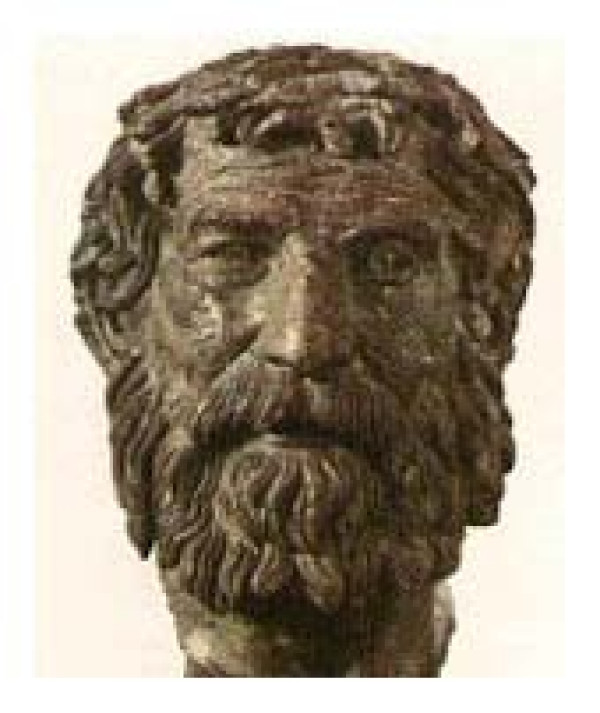
**A portrait of Empedocles, a Greek pre-Socratic philosopher**.

**Figure 6:**
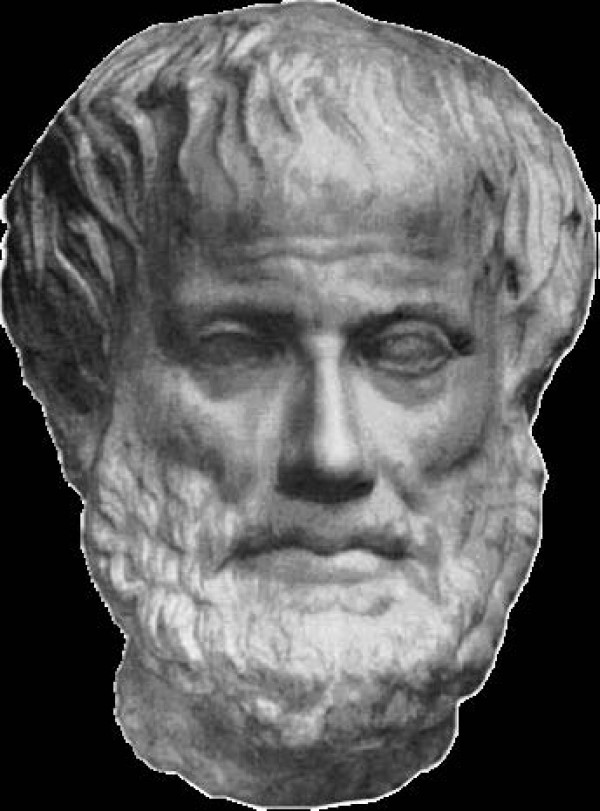
**Portrait of Aristotle, a Greek philosopher, student of Plato and teacher of Alexander the Great**.

Classical Greek philosophers were not an exemption and there are a lot of references in their work about the origin and function of the spine. Plato (427-347 BC) (Figure 4), who influenced the disciplines of philosophy, psychology, logic, and politics, through his conceptualization that mathematics is the life force of science, implicated biomechanics in function of the spine. However he believed that a divine intervention contributed to the creation of the flexible spine [3]. In contrary, Empedocles (490-430 BC) (Figure 5) thought that the vertebrae are initially unified (rigid spine) and subsequently this solid osseous column brake down (segmented) into pieces as a result of movements of the body [4]. Aristotle (384-322 BC) (Figure 6), who was the most prominent research scientist in ancient Greece, lived in a period when athletics, sports, and gymnastics was part of philosophy of developing the human being as a whole to optimize functional capacity and harmony. Álthough Aristotle's studies were not directly related to the spine, in his treatises, *Parts of Animals, Movements of Animals*, and *Progression of Animals*, he described the action of the muscles and subjected them to geometric analysis for the first time. For this work, he is considered as a biomechanist and the father of kinesiology [5]. Aristotle defined the act of "muscular flexion" as a change from a straight line to an angle and noted that without this "flexion," there could not be forward progression, such as walking and swimming. This implied, for the first time, the thought or conceptualization of transformation of rotatory into translational motion.

**Figure 7:**
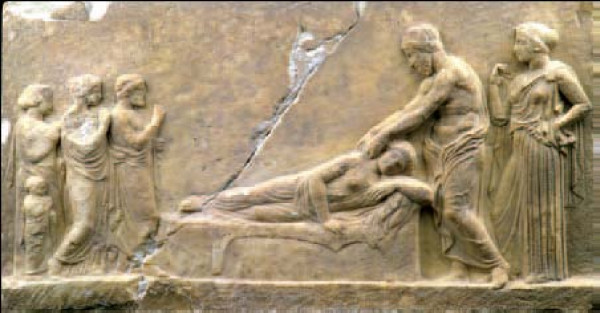
**Asclepios, the God of health, while examining a patient**.

Medicine was practiced at Asclepions, which were temples dedicated to Aesculapius, the god of health (Figure 7). A priest-physician was responsible for examinations and treatments. Treatment modalities in the Asclepions included hydrotherapy, physiotherapy, hygienic rule, diet, well-known drug therapies, and minor surgical procedures. It is assumed, but not proven, that minor spinal disorders were treated in these facilities.

Physicians in ancient Greece had a remarkable knowledge of anatomy, although dissection of human bodies was prohibited. They derived their knowledge from cadavers in battlefields, from observations of athletes exercising in the gymnasiums, and from dissections of animals.

## Spinal deformities in ancient Greece

### Spinal Deformities in the work of Hippocrates and Galen

**Figure 8:**
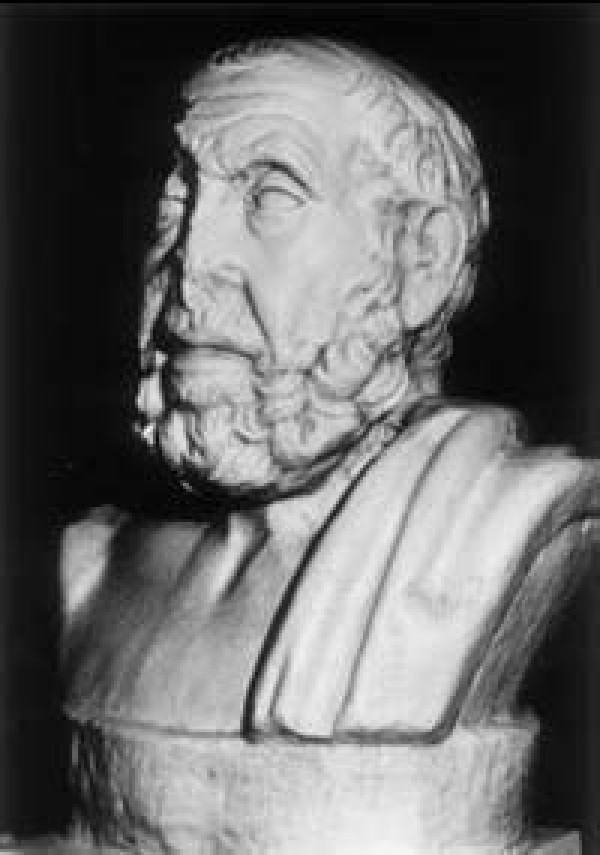
**Hippocrates the Koan**.

**Figure 9:**
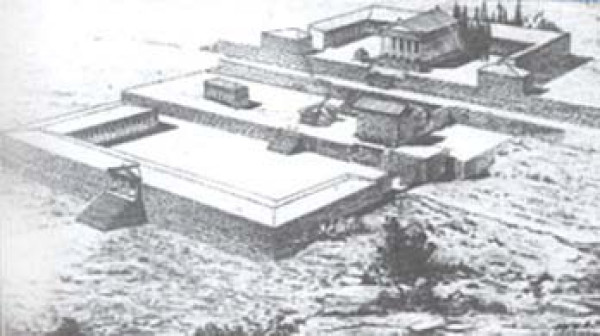
**A graphic illustrating the Asklepion of Kos**.

**Figure 10:**
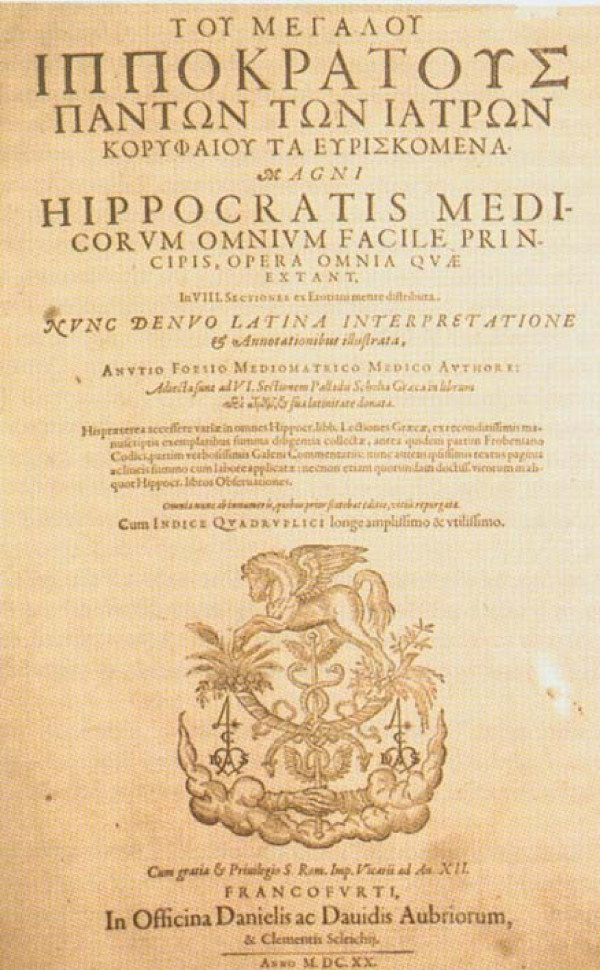
**An illustration showing one of the numerous editions of "Corpus Hippocraticum"**.

The most well-known physician of antiquity was Hippocrates (460-370 BC) (Figure 8). He was born on the Greek island of Kos, where he studied and practiced medicine in the Asclepion of Kos (Figure 9). Hippocrates through scientific thought freed medicine from the "influence" of supernatural spirits and transformed it from an empirical and religious art to a science and today he is recognized as the founder of scientific medicine. Hippocrates' wrote almost as many as 60 medical books, which are included in the *Hippocratic Collection *(*Corpus Hippocraticum*) (Figure 10). Information about spinal deformities is incorporated in his books, namely, *On fractures (Περί αγμών, "Peri agmon")*, *On articulations (Περί άρθρων, "Peri Arthron")*, *Mochlikon (Μοχλικός, "Mochlikos"), On Nature of Bones (Περί οστέωνφύσιος, "Peri osteon physios") *and *On Places in Man (Περί τόπων των κατ' ανθρώπων, "Peri topon ton kat anthropon")*. His work is dominated by the principle of accurate observation and logical reasoning which leads to accurate conclusions for the diseases [6,7].

**Figure 11:**
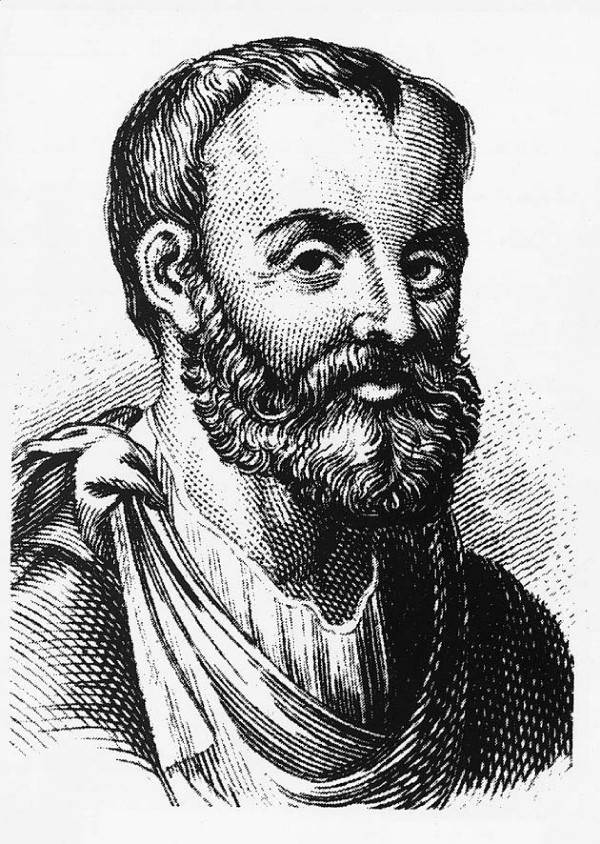
**Galen of Pergamon**.

Galen of Pergamon (130–200 AD) (Figure 11), another eminent Greek physician, who initially was the physician of gladiators, wrote numerous medical books in Greek language, but unfortunately only 118 treatises were saved from a fire in Rome, where he served as the physician of the emperor, Marcus Aurelius. He worked as a surgeon and anatomist and was the founder of experimental physiology and embryology [8]. Today, the reference collection of Galen's works is a 22-volume edition of C. G. Kühn (Lipsiae 1821–1833), which offers the Greek text with a Latin translation. Many of his texts has never been translated into modern languages, therefore they are inaccessible to the public. Even if we were to eliminate the writings of the *Corpus Hippocraticum*, Galen's exceptional output would still represent more than 80% of all surviving medical writings of antiquity [8]. In his medical books there are a lot comments on the Hippocratic books. Information about spinal deformities are contained in his books under the titles *Hippocrates' Peri arthron and Four Comments on It («Ιπποκράτους, το περί άρθρων βιβλίον και Γαληνού εις αυτό υπομνήματατέσσεραα»), Three Comments on Hippocrates' Peri agmon («Εις το Ιπποκράττους, περίαγμών»), On Bones for beginners («Περί οστών τοις εισαγομένοις») and On the Usefulness of the Parts of the Body («Περί χρείας των εν ανθρώπον σώματι μορίων»)*.

### Anatomy and biomechanics of the spine

Hippocrates considered knowledge of the spinal anatomy essential to physicians: *"One should first get a knowledge of the structure of the spine; for this is also requisite for many diseases" *[9]. According to legend, the "great" Hippocrates used his knowledge of human anatomy to create a copper copy of the skeleton, which he offered to the oracle at Delphi.

In his book *On Nature of Bones*, Hippocrates describes that the function of the bones and particularly of the spine is to maintain the erect position of man and to form the shape of the human body [10]. He describes the anatomy and the diseases of the spine and suggests treatments for patients with spinal deformities. This is the first systematic presentation of anatomy and pathology of the spine in medical history. He realized that the spine was held together by means of intervertebral discs, ligaments, and muscles, permitting him to describe the normal curvatures of the spine [11]. This remarkable knowledge of anatomy derived from cadavers in battlefields, from observations of athletes exercising in the gymnasiums, and from dissections of animals, because dissection of human bodies was prohibited. These were first performed many years later by Herophilus of Chalcedon (335-280 BC) [4].

Galen agreed with Hippocrates' principle that a good knowledge of spinal anatomy is an absolute prerequisite for its treatment and provides a comprehensive description of the muscles, vertebrae, discs, ligaments, meninges, and spinal cord as well as a detailed description of the normal spinal curvatures [11]. Galen studied anatomy in Alexandria of Egypt, the most important center for anatomical studies in antiquity, thanks to the Herophilus who carried out human dissections.

In Galen's book *On the Usefulness of the Parts of the Body *[12] there is a description of the anatomy of the spine which has few differences from those found in contemporary medical texts [11]. His anatomic doctrines affected medicine for more than 1200 years, until the studies of Vesalius altered knowledge. In the same book he pledges that nature's wisdom formed the structure of the spine and wrote: *"Nature creates nothing without a purpose"*. He wrote that the vertebrae are safely bound together in their anterior surface and articulated in the back. The ventral part of the spine provides for the harmony in spinal motion, while the dorsal section ensures stability and safeness and continues: *"Nature, tends to keep everything in motion and at the same time aims at the security of its components. The vertebral column exemplifies how these two more or less opposite trends can keep in balance. If the spine was a single, rigid bone, then it would be invulnerable but also inflexible like a statue. In that case man would have been deprived of motion, which is the vital feature of life. On the other hand, a spine consisting of many small parts would be more flexible, but the unavoidable consequence of this flexibility would have been its vulnerability. The number of the existing vertebrae is the ideal as it allows the spine to bend in a circular rather than in an angular manner thus avoiding the injury of the spinal cord"*.

### Description of normal and abnormal spinal curves

**Figure 12:**
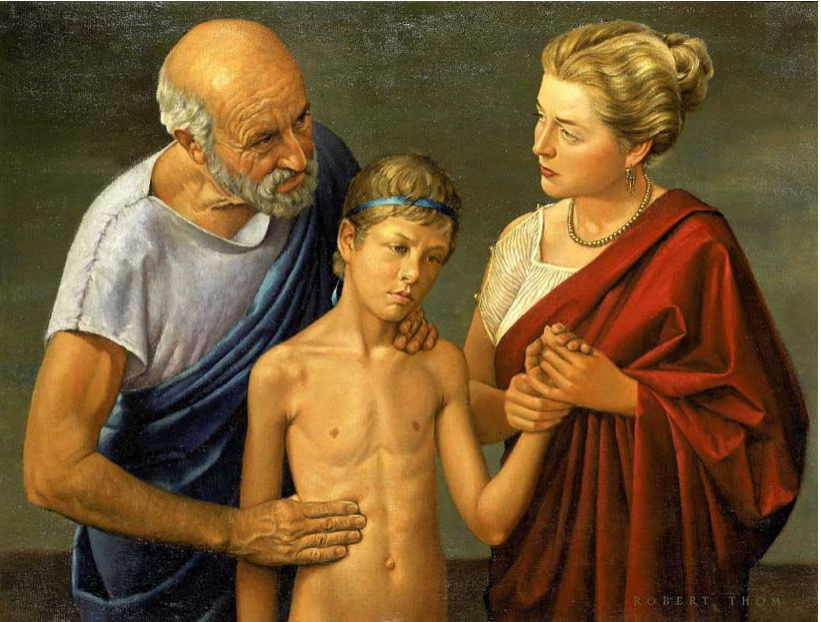
**Hippocrates examining a child, a painting by Robert Thom, 1950's**.

In his books *On articulations *and *Mochlikon*, Hippocrates describes the normal curves of the spine in a most articulate manner (Figure 12). He classified the spinal vertebrae into three groups. The first group consisted of the vertebrae lying above the level of the clavicle. The C2 and the great vertebra (which corresponds to either C1 or C7) were in this group. The second group included the thoracic spine; the third group consisted of the five vertebrae between the chest and the pelvis. He uses the term "ithiscolios", which indicates that the spine is straight in the coronal, but curved in the sagittal plane [9]. He believed that kyphosis of the thoracic spine is mostly deceptive, because the spinous processes are higher than those in the overlying and the underlying sections of the spine [9,13]. Lordosis of both the lumbar and cervical spine is a normal feature. Although he does not consider the sacral vertebrae and the coccyx as parts of the spine, he refers to both when he describes the normal curves of the spine.

In Hippocrates' treatise *On Articulations*, one of the most important surgical texts of the entire Hippocratic Collection, Hippocrates classifies diseases of the spine in five groups and presents the etiology, the clinical manifestations, and the management of these diseases. The five groups of spinal diseases which introduced by Hippocrates are, a) kyphosis as a result of either a disease (non traumatic) or spinal injury (traumatic), b) scoliosis, c) concussion ("seisis") which means burst fractures, d) dislocations of the vertebrae and e) fractures of the spinous processes.

Galen described four types of spinal deformities, namely kyphosis when they spinal column moves backward, lordosis when it moves forward, scoliosis when it moves to the side and succussion, where there is no spinal deformity but the intervertebral articulations still have moved. He comments that Hippocrates used the term scoliosis to describe all spinal deformities [8]. He agreed with Hippocrates that these deformities can be caused by the presence of tuberculous nodes in the lung which usually leads to kyphosis, but also lordosis or scoliosis, by a spinal injury due to a fall either on the hips or on the shoulders, as a result of aging and fatigue of the spine and because of painful conditions. The mechanism of the deformity, according to Galen, is the formation of tuberculous nodes next to the vertebrae as well as intervertebral ligament shrinkage and pulling of the vertebrae toward the nodes. Depending on the number and location of the nodes, all three types of deformities can be produced [8].

Description of spinal injuries is beyond the aim of the present study. In the following paragraphs, only the pathology and treatment of spinal deformities is discussed.

### Description of Spinal Deformities

#### Non traumatic Kyphosis

Hippocrates described four different causes of nontraumatic kyphosis, namely tuberculous spondylitis, epilepsy (the sacred disease), congenital or aquired bilateral dislocation of the hip, but it may also appear in healthy people. In his treatise *On articulations *he writes "*Curvature of the spine occurs even in healthy persons in many ways, for such a condition is connected with its nature and use; and besides, there is a giving way in old age and on account of pain"*.

In his book *On Places in Man*, Hippocrates divided tuberculous spondylitis into two categories. Galen endorses the Hippocratic division of the disease in these two categories. In the first category, the curvature of the spine is formed above the attachment of the diaphragm and are thought to be incurable, while in the second, the hump is situated below this level. The skeletal changes concern the hips and the spine: "*The hips are still more attenuated in such cases than where the hump is high up; yet the spine as a whole is longer in these than in high curvatures" *[9].

He describes spinal and thoracic deformity in a remarkable way: *"the ribs do not enlarge in breadth, but forwards, and the chest becomes pointed instead of broad; the patients also get short of breath and hoarse, for the cavities which receive and send out the breath have smaller capacity. Besides, they are also obliged to hold the neck concave at the great vertebra that the head may not be thrown forwards" *and continuous *"...these patients have also, as a rule, hard and unripened tubercles in the lungs; for the curvature and contraction is in most cases due to such gatherings, in which the neighbouring ligaments take part" *[9].

The deformity of the spine is more pronounced in patients who have not reached puberty, implicating the role of growth in development of the deformity: *"When hump-back occurs in children before the body has completed its growth, the legs and arms attain full size, but the body will not grow correspondingly at the spine; these parts are arrested in their development" *[9]. In adults, the disease has a more benign course, because the growth of the body already has been completed: *"When curvature comes on in persons whose bodily growth is complete, its occurrence produces an apparent crisis in the disease then present. In time, however, some of the same symptoms found in the younger patients show themselves to a greater or lesser degree; but in general they are all less malignant" *[9].

Twenty-three centuries after Hippocrates described tuberculus spondylitis, Percivall Pott (1714–1788), a famous British surgeon, described spinal tuberculosis in his work *Remarks on the Kind of Palsy of the Lower Limbs Which is Frequently Found to Accompany a Curvature of Spine*. Today, tuberculous spondylitis is known as "Pott's disease" [14].

#### Traumatic Kyphosis

Hippocrates describes the pathology of post-traumatic kyphosis, commonly caused by falling on the shoulder or buttock in his book *On Articulations *and explains why the spinal cord usually is not injured:

*"...in the curvature, one of the vertebrae necessarily appears to stand out more prominently, and those on either side less so. It is not that one has sprung out to a distance from the rest; but each gives way a little, and the displacement taken altogether seems great. This is why the spinal marrow does not suffer from such distortion, because the distortion affecting it is curved and not angular"*, [9] and concludes that this condition has low mortality: *"(Deviations) in the form of a hump are not as a rule injuries which cause death, retention of urine, or loss of sensation ... for external curvature does not stretch the ducts which pass down the body cavity, nor does it hinder free flow" *[9].

#### Scoliosis

In the Hippocratic works, the term "scoliosis" has a general meaning and applies to almost every kind of spinal curvature, including those spinal deformities resulting from injuries of the vertebrae with or without dislocation of the vertebral bodies. When the term is restricted to its contemporary meaning, then little information can be derived from the Hippocratic texts [15]. Hippocrates mentions two possible causes of the diseases: *"due to 'gatherings' (probably tuberculous abscesses) on the inner side of the spine and postural *– *while in some cases the positions the patients are accustomed to take in bed are accessory to the malady"*. Although Hippocrates promises to discuss the issue of scoliosis together with chronic diseases of the lung in *On Articulations*, this commitment was never fulfilled, at least in the Hippocratic texts preserved until today. Galen believes that this may be the result of a loss of some of the Hippocratic treatises, in which there might have been references to this disease. He describes a strange case of scoliosis of the cervical spine, which is related to severe sore throat in the second book of *Epidemics (Επιδημίαι, "Epidimiae")*. [16]

#### Treatment of Spinal Deformities

When Hippocrates refers to management of the spinal deformities he makes no distinction between the various types; thus, the methods presented in his books apply to almost every kind of spinal curvature.

Hippocrates recommended diet and extension for the treatment of scoliosis. Spinal manipulation as a treatment for spinal deformities was widely practiced at the time of Hippocrates.

He was the first who invented devices based on principles of axial traction and three points correction for correction of curvatures of the spine and the management of spinal diseases. The devices used by Hippocrates for treatment of spinal deformities were the Hippocratic ladder, the Hippocratic board and the Hippocratic bench. Although Hippocratic books do not contain illustrations, these are provided by Apollonius of Kitium (1st century BC), who commented on the techniques presented by Hippocrates in *On Articulations*. These fine illustrations are preserved in a Florentine surgical manuscript (*Laurentianus 74. 7*, 9th century AD) [17].

#### Hippocratic ladder

**Figure 13:**
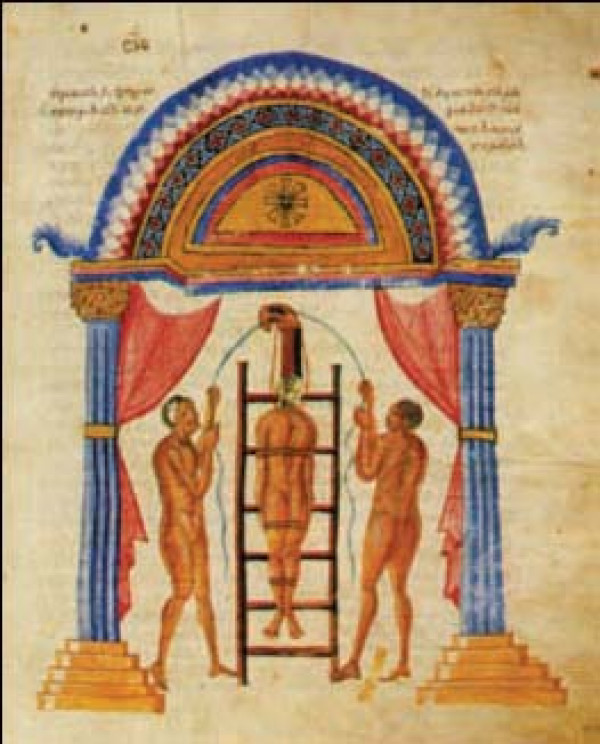
**The Hippocratic ladder for correction of spinal deformities with the head pointing upwards**. From the illustrated comments of Apollonius of Kitium on the Hippocratic treatise *On Articulations*. Bibliotheca Medica Laurenziana, Florence.

**Figure 14:**
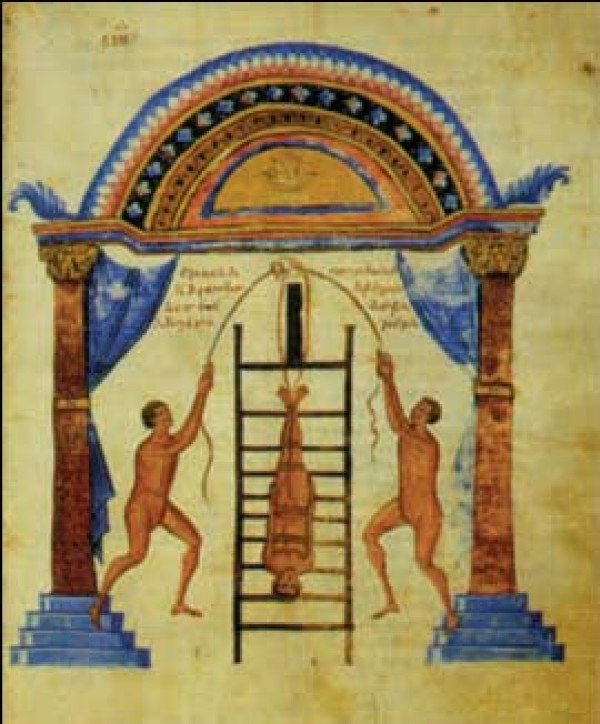
**The Hippocratic ladder for correction of spinal deformities with the head pointing downwards**. From the illustrated comments of Apollonius of Kitium on the Hippocratic treatise *On Articulations*. Bibliotheca Medica Laurenziana, Florence.

Hippocratic ladder was developed to reduce spinal curvatures. To achieve reduction, the patient must undergo succussion (shaking) while being tied on a ladder, in erect position if the hump is near the neck (Figure 13) or with the head downwords if the hump lies in a lower level (Figure 14). The weight of the trunk and the limbs act as the pulling force, which straightens the spine. Hippocrates describes the board as the most efficient method for the correction of spinal deformities because the physician can easily control the forces exercised on the spine and these forces are exerted in accordance with nature: *"for the pressure forces the protruding parts into place, and the extensions according to nature draw the parts that have come together" *[9]. The method is so powerful that either traction or pressure alone is enough to accomplish reduction, but *"...it is difficult to perform extension at the neck, because the patient might suffocate"*. [9]

*"If one desires to do succussion, the following is the proper arrangement. One should cover the ladder with transverse leather or linen pillows, well tied on, to a rather greater length and breadth than the patient's body will occupy. Next, the patient should be laid on his back upon the ladder; and then his feet should be tied at the ankles to the ladder, without being separated, with a strong but soft band. Fasten besides a band above and below each of the knees, and also at the hips; but the flanks and chest should have bandages passed loosely round them, so as not to interfere with the succussion. Tie also the hands, extended along the sides, to the body itself, and not to the ladder. When you have arranged things thus, lift the ladder against some high tower or house-gable. The ground where you do the succussion should be solid, and the assistants who lift well trained, that they may let it down smoothly, neatly, vertically, and at once, so that neither the ladder shall come to the ground unevenly, nor they themselves be pulled forwards. When it is let down from a tower, or from a mast fixed in the ground and provided with a truck, it is a still better arrangement to have lowering tackle from a pulley or wheel and axle *[9].

#### Hippocratic board

**Figure 15:**
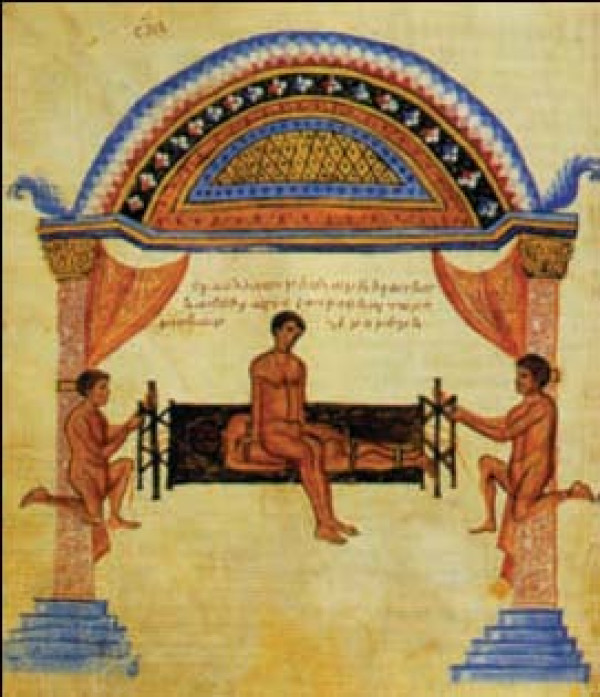
**An illustration of Hippocratic board by Apollonius of Kitium showing the correction of a spinal deformity**. To reduce the hump, the physician or a trained assistant uses his hands, his foot, or even his whole body to press it, while traction is applied. Bibliotheca Medica Laurenziana, Florence.

**Figure 16:**
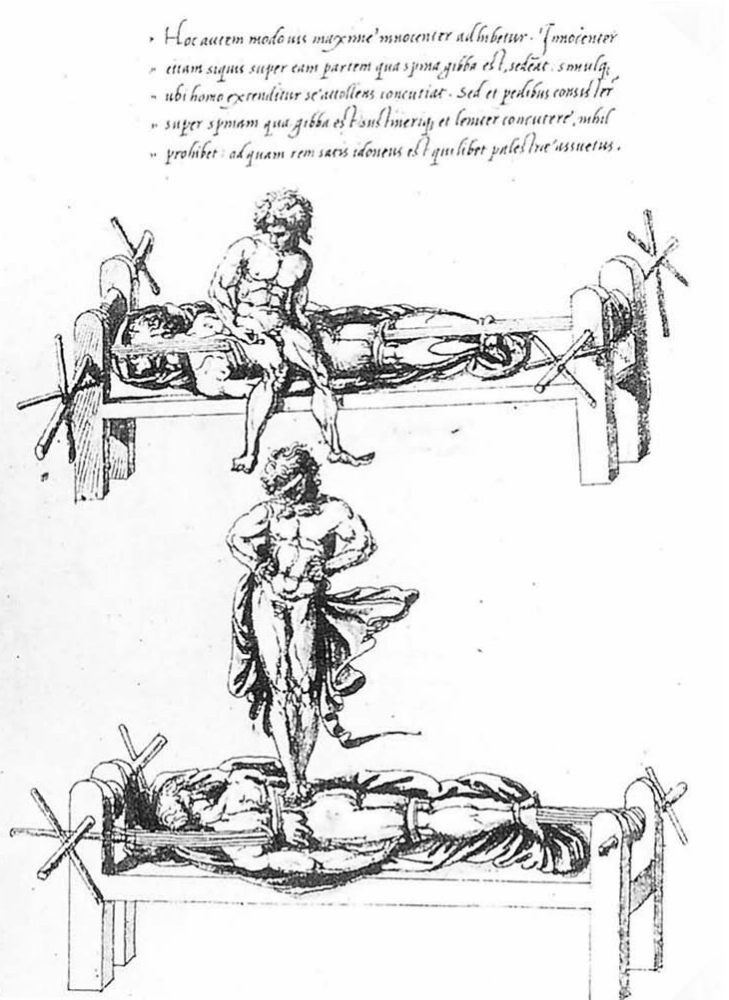
**A drawing attributed to Vidus Viceus (16^th ^century AD) showing correction of spinal deformity on the Hippocratic board**. Paris National Library.

Hippocratic board is another device to manage spinal curvatures. The technique which was recommended was simultaneous traction of the spine and the manual application of focal pressure over the kyphotic area (Figures 15, Figure 16): *"But the physicians, or some person who is strong, and not uninstructed, should apply the palm of the hand to the hump, and then, having laid the other hand upon the former, he should make pressure, attending whether this force should be applied directly downward, or toward the head, or toward the hips ... and there is nothing to prevent a person from placing a foot on the hump, and supporting his weight on it, and making gentle pressure; one of the men who is practiced in the palestra would be a proper person for doing this in a suitable manner .. " *[9].

**Figure 17:**
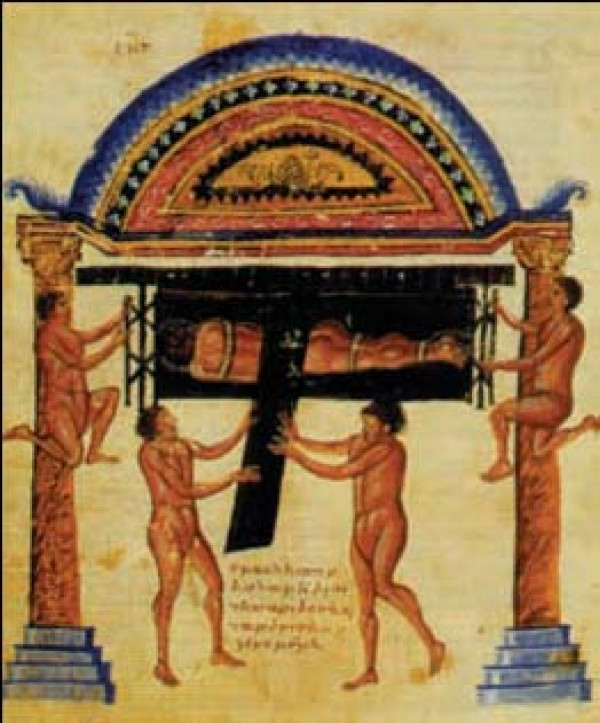
**An illustration of Hippocratic board by Apollonius of Kitium showing correction of a spinal deformity by applying stronger force to restore the anatomy of the spine, by using a plank**. One end of this plank is adjusted to an incision made in the wall or in the post embedded in the ground. With the hump lying below the plank, one or two assistants press down its opposite end. Bibliotheca Medica Laurenziana, Florence.

**Figure 18:**
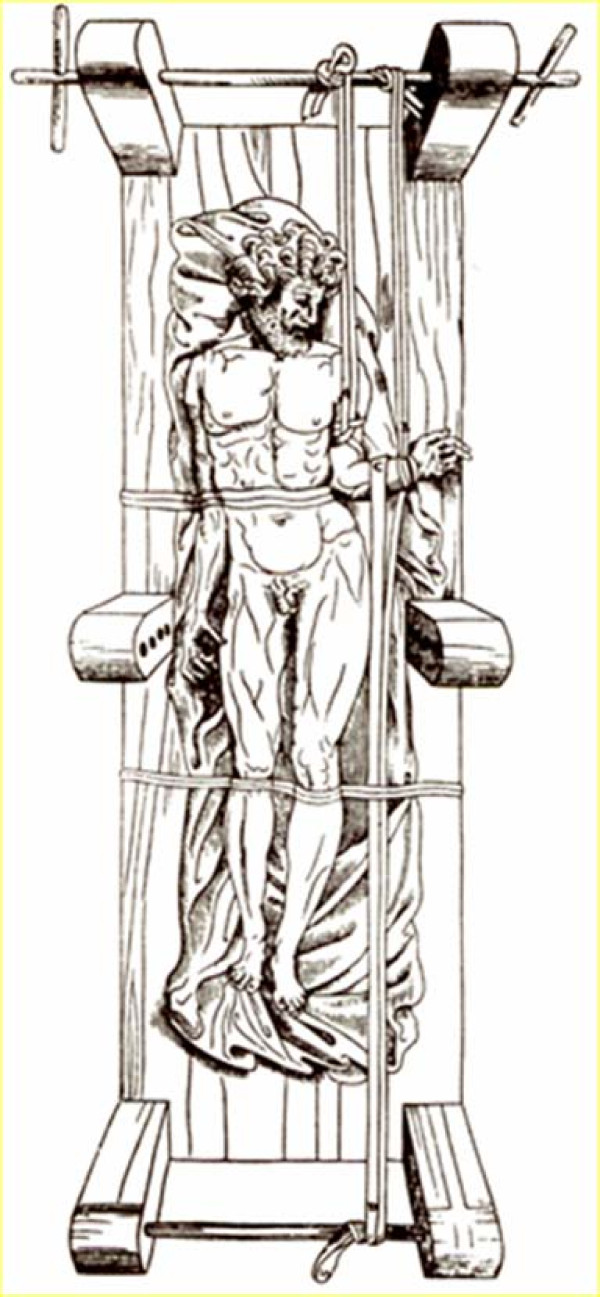
**A schematic representation of the application of corrective forces for spinal deformities by the use of straps and bands, properly adjusted on the Hippocratic board**.

For patients where stronger forces are required Hippocrates recommended (Figure 17): "*The apparatus for forcible reduction should be arranged as follows. One may fix in the ground a strong broad plank having in it a transverse groove. Or, instead of the plank, one may cut a transverse groove in a wall, a cubit above the ground, or as may be convenient. Then place a sort of quadrangular oak board parallel with the wall and far enough from it that one may pass between if necessary; and spread cloaks on the board, or something that shall be soft, but not very yielding. Give the patient a vapour bath if possible, or one with plenty of hot water; then make him lie stretched out in a prone position and fasten his arms, extending them naturally, to the body. A soft band, sufficiently broad and long, composed of two strands, should be applied at its middle to the middle of the chest, and passed twice round it as near as possible to the armpits; then let what remains of the (two) bands be passed round the shoulders at each side, and the ends be attached to a pestle-shaped pole, adjusting their length to that of the underlying board against which the pestle-shaped pole is put, using it as a fulcrum to make extension *(Figure 18). *A second similar band should be attached above the knees and above the heels, and the ends of the straps fastened to a similar pole. With another soft, strong strap, like a head-band, of sufficient breadth and length, the patient should be bound strongly round the loins, as near as possible to the hips. Then fasten what is over of this band, as well as the ends of both the other straps, to the pole of the foot end; next, make extension in this position towards either end simultaneously, equally and in a straight line *[9].

**Figure 19:**
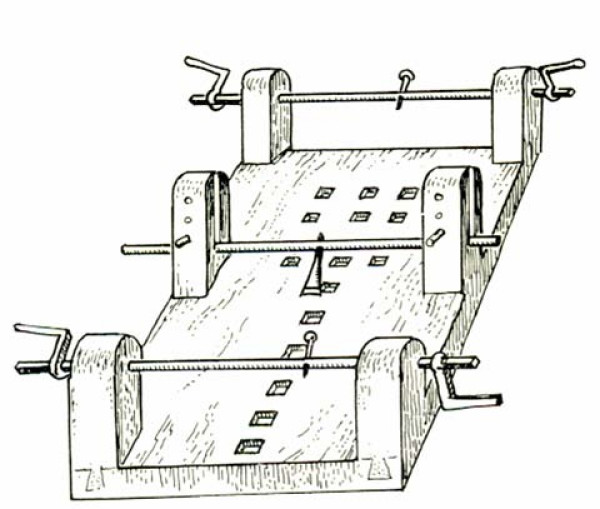
**The Hippocratic board with the addition of the third bar by Orebasius**.

Oribasius (325–400 AD), a Byzantine physician, added a bar to the Hippocratic board and used it for reduction of both spinal traumas and deformities [3] (Figure 19).

**Figure 20:**
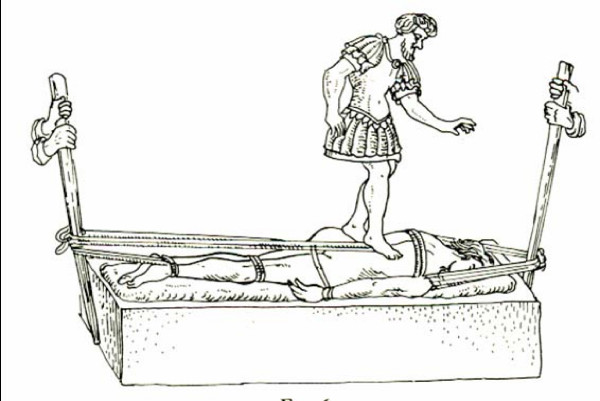
**A drawing showing Galen's method of correction of spinal deformity on a device similar to the Hippocratic board by applying pressure on the patient's back**.

**Figure 21:**
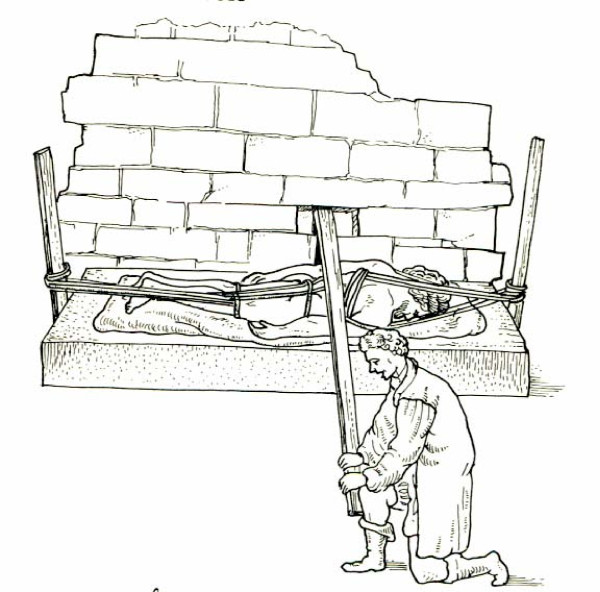
**A drawing showing Galen's method of correction of spinal deformity on a device similar to the Hippocratic scamnum by applying pressure with the use of a board attached in the wall**.

Galen recommended the use of the Hippocratic board (Figures 20, Figure 21) for traumatic deformities according to which correction of the curvature is achieved through a combination of traction and pressure and the Hippocratic ladder for kyphotic deformities although he expresses his doubts on the effectiveness of this technique [18].

Paulus of Aegina (625–690 AD), although lived in the Byzantine period, he is considered the last physician of Greek antiquity. He collected doctrines of the antique period in his seven-volume encyclopedia. In his medical practise, he used the Hippocratic board for management of spinal deformities and also emphasized in the use of orthoses in spinal trauma and deformities [3].

#### Hippocratic scamnum

**Figure 22:**
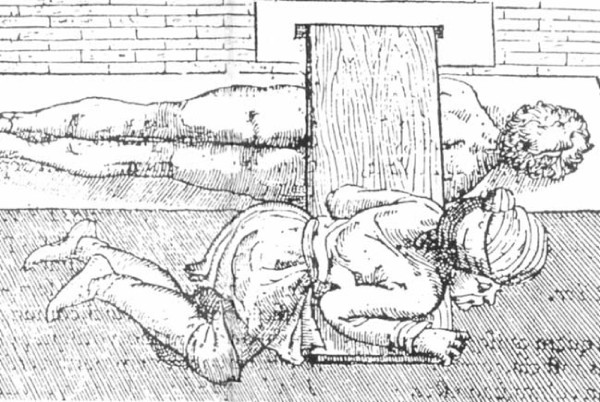
**The Hippocratic scamnum**.

The third device for the management of spinal deformities was the Hippocratic scamnum (Figure 22): *"But the most powerful of the mechanical means is this; if the hole in the wall, or in the piece of wood fastened into the ground, be made as much below the man's back as may be judged proper, and if a board, made of lime-tree, or any wood, and not too narrow, be put into the hole, then a rag, folded several times or a small leather cushion, should be laid on the hump ... when matters are thus adjusted, one person, or two if necessary, must press down at the end of the board, while others at the same time make extension and counter-extension along the body, as formerly described" *[9].

#### Other treatment methods

Hippocrates often experimented with new methods. One of his unsuccessful attempts being described in *On Articulations*. An advanced treatment method involve placing the patient supine on the Hippocratic board and trying to fill with air a leather sack positioned under the patient's spine, which, when attempted by Hippocrates, it proved to be unsuccessful. Hippocrates in *On Articulations*, conceived the idea of transabdominal correction of spinal deformities, although he was reluctant to perform such an operation on a living patient [15].

#### Ethics in management of spinal deformities

Hippocrates refers to the ethical issues arising from the use of methods of reduction. He warns patients against charlatans and incompetent practitioners who not only demonstrate treatment methods to impress their audience but also use such forcible maneuvers for harm and not for healing: *"Wherefore succussion on a ladder has never straightened anybody, as far as I know, but it is principally practiced by those physicians who seek to astonish the mob- for to such persons these things appears wonderful. For example, if they see a man suspended or thrown down, or the like; and they always extol such practices, and never give themselves any concern whatever may result from the experiment, whether bad or good. But the physicians who follow such practices, as far as I have known them, are all stupid" *[9].

## Epilogue

**Figure 23:**
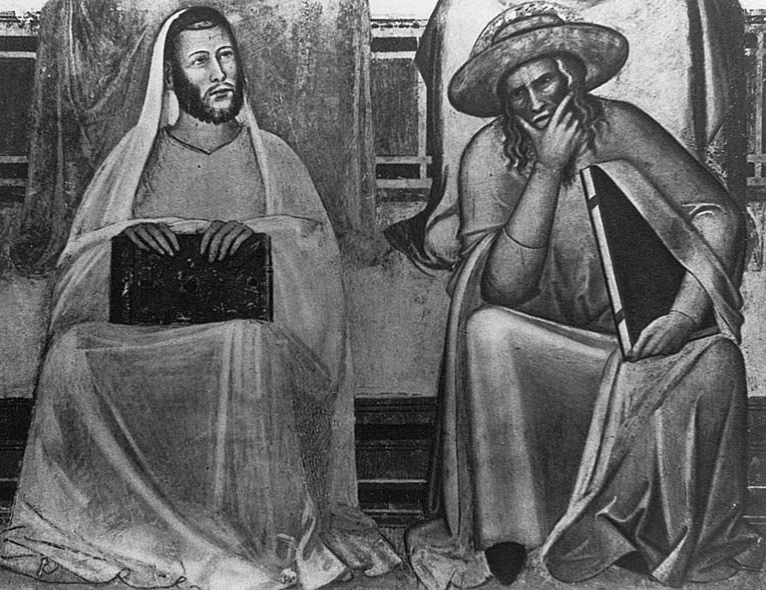
**Hippocrates and Galen**. Fresco of Taddeo Gaddi, Santa Maria Novella, Florence, Italy.

Hippocrates and Galen, who's writings were in harmony with Christian faith (Figure 23) attempted to describe spinal deformities, to discuss their pathology and to recommend treatment modalities based on fundamental principles which are still adopted today. Their work is the first scientific approach of understanding and treating spinal deformities. Medicine made a significant progress since ancient times, but there are always new things to be discovered. Hippocrates writes in his book *On Ancient Medicine*: *"Medicine has always existed since the beginning of time. The road has been revealed to us, and many good things have been discovered along the way. The rest remain to be discovered if one based on what is already known is capable enough to ask for more"*.

## Competing interests

The authors declare that they have no competing interests.

## Authors' contributions

EV conceived the idea of the presented study, performed the literature review, and performed the manuscript drafting. TBG and AK contributed in manuscript drafting. All authors have read and approved the final manuscript.
